# The Distribution Committee

**Published:** 1914-08-08

**Authors:** 


### METROPOLITAN HOSPITAL SUNDAY FUND.

### The Distribution Committee.

A meeting of the Council of the Fund was held at the Mansion House on Friday, July 31. In the absence abroad of the Lord Mayor, the President and Treasurer of the Fund, Sir Wm. S. Church, Bart., M.D., K.C.B., was voted to the chair, and there were present: The Right Hon. Viscount Knutsford, Lord Meath, Rev. W. Hardy Harwood, Rev. Prebendary Perry, Rev. Prebendary Ingram, Canon Pearce, Dr. E. Parker Young, Very Rev. Monsieur Carton de Wiart, Ex-Sheriff Cooper, and the Revs. E. S. Hilliard, Andrew Crombie, A. A. Harland, and A. Hall.

The minutes of the last meeting having been read and approved, the Secretary, Mr. James, announced that letters of regret at their absence had been received, among others, from the Bishop of Islington, the Dean of Westminster, the Chief Rabbi, Sir John Bell, Sir Alfred Pearce Gould, and Sir Henry Burdett.

### Resignation of Sir John Bell.

The Secretary read the following letter, which had been received from Sir John Bell, dated from his country seat on July 14:

"My Dear Lord Mayor,—I am desirous of retiring from the Chairmanship of the Hospital Sunday Fund, a position which has always been held by a past Lord Mayor, or, at all events, by an Alderman. I am leaving England on the 25th inst. for treatment to my arm (which, I am sorry to say, still causes me trouble), and, in consequence, shall not be present at the next Council meeting. Will you, therefore, please make the announcement at the opening of the proceedings, and express my thanks to the whole of the members for their uniform kindness and consideration during all the years I have been a member, and especially while acting as Vice-Chairman?—Kindest regards, yours very truly, J. C. Bell."

Lord Knutsford moved that the Council receive the resignation with regret, and pass a very cordial vote of thanks to Sir John Bell for all that he had done for the Council and its work. Sir John had been a very regular attendant at the meetings, and on many occasions had ably presided.

The Chairman added that for many years Sir John Bell had been a member of the Distribution Committee, and a very regular and active participant in its operations. He supported the proposal of Viscount Kuntsford, that a letter be sent to Sir John expressing the Council's regret.

This was unanimously carried.

The Chairman pointed out that it would be necessary to give due notice concerning the appointment of a Vice-Chairman before that could be carried out.

Viscount Knutsford moved that Sir Ernest Tritton, Bart., be asked to assume the duties of Vice-Chairman of the Fund temporarily, until the holding of the meeting at which a successor in the post could be formally appointed. Sir Ernest Tritton seemed obviously to be the right man, for he had been in daily touch with the Fund during the whole of the late Sir Edmund Currie's illness, and, with the able assistance of the present Secretary, did much in reorganising the staff.

This was seconded and unanimously approved.

### Obituary.

The deaths of the following members of the Council were recorded: Rev. Canon Rhodes Bristow, who had been a member 33 years; Robert Gray, Esq., 22 years; Rev. Canon McCormick, 18 years; Rev. Prebendary Shelford, 23 years; Rev. C. H. Grundy, 18 years; Sir E. Durning-Lawrence, Bart., 11 years; and John Lile, Esq., 5 years.

The Chairman said that the Distribution Committee felt the loss of Mr. Gray so keenly that they at once communicated their sympathy to the members of his family.

### Proposed Co-ordination of District Nursing Associations.

The Chairman said he believed that most of the members present had heard of the conferences which had been held as to the district nursing of the Metropolis, but before his action would be understood it was necessary for him to make a short statement. In June 1913 Mr. Burns called a conference of a number of different bodies and people interested in district nursing, and in some other charitable matters, and forwarded an invitation to the Hospital Sunday Fund to send a representative to the conference. As Chairman of the Distribution Committee, it fell to him, Sir William, to go. It was well known that the Fund, for a considerable number of years, had given 2½ per cent. of the total amount collected to some thirty-two district nursing associations in the Metropolis. Of course the Distribution Committee made inquiries, because it insisted that the nurses in such bodies should be fully qualified persons. Probably the knowledge of this fact on the part of the Local Government Board constituted the reason for the Fund being asked to send a representative to the conference. Mr. Burns, at the meeting, made a speech, and the outcome of the meeting was the appointment of a provisional committee to consider the whole question. That occurred in 1913. This year a second conference had been convened, and it met on June 12, when the provisional committee presented their report, on which evidently a good deal of labour had been expended, for it embraced much information. It also sketched out a kind of organisation for a central bureau, to which the various nursing associations should be more or less affiliated, the idea evidently being that the bureau should be to some extent a controlling body over them. The conference in June last was addressed by Mr. Samuel, who stated that, although there was no mention of it in the Budget, the Chancellor of the Exchequer intended to grant a quarter of a million for nursing purposes during the year, the grant appearing in the Supplementary Estimates. Mr. Lloyd George had made only brief mention of the devotion of so large a sum to nursing. Two reports were presented to the conference, a majority report and a minority report. Sir Arthur Downs moved at a meeting that a Central Council for London should be established, consisting of representatives of those engaged in district nursing and of others interested in the question, with the object of so systematising the arrangements for district nursing that there should be adequate provision throughout the county meaning thereby the whole County of London. That was carried. Sir Arthur Downs then moved a second resolution providing that the council be composed of a president, vice-president, and members, and that such council should be so constituted as to include representatives of the churches, public authorities, charitable and philanthropic agencies, friendly societies, the medical profession, the nursing profession, district nursing associations, and other persons interested in the matter. The question was an important one, because the grants of the Fund were made only to nursing associations which made use of fully-qualified nurses. It was necessary to report the matter to this Council, because it had now been invited to send a representative to serve on the council now being established.

Viscount Knutsford said the difficulty appeared to be that, though they would be glad for Sir William Church to represent the Council on the council of the nurses' organisation, that grants from the Fund were made only to those nursing establishments which sent out duly trained and qualified nurses. And it was increasingly difficult to get fully-trained nurses to take up district nursing, and he did not feel sure that it was quite the province of the fully-trained nurse. It was essential to have a fully-trained nurse to nurse in a district where nurses were not easily got, and residents in the country knew that excellent work was being done in villages and districts by nurses who would not be described as fully trained. But their work was much lessened by the fact that the poor who were seriously ill could go into the hospitals, and there was always a doctor at hand, so that the work of the nurse consisted largely in nursing chronics, people in receipt of Poor-Law relief, and those who had received small injuries, which duties did not necessarily call for a fully-qualified nurse. He doubted whether the newly-established council would restrict their grants to fully-trained nurses. He thought that any district which employed nurses not fully trained should be under the supervision and control of a Queen's (a fully-qualified) nurse.

The Chairman said it was proposed that the work would be done by an executive committee, and the general council would, he believed, be only a supervising body. He had no particular wish to be a member of that body, and he did not think the matter was at all urgent. The difficulty alluded to by Lord Knutsford was dwelt upon at the conference at considerable length, and his personal preference was for the minority report. Many of the questions, on being gone into, proved very difficult ones. For instance, a burning question at the present time was that of the appointment of whole-time nurses as nurses in connection with schools.

Viscount Knutsford proposed that Sir William Church be asked to join the central body, and it was agreed to.

### The Amount Distributed.

The Chairman said he was willing to go in obedience to that resolution.

He then proposed : " That the report of the Committee of Distribution for the year 1914 be, and is hereby, approved; and that the awards thereby recommended be paid forthwith." The amount distributed is £59,500, including dividends which would be paid on August 5. In answer to a question, he said that as money was still continuing to come in, and was likely to for some time, the exact amount could not yet be stated, but in the year's report the amounts from the various churches were always given.

The Rev. Hardy Harwood seconded the motion, and it was unanimously carried.

The Rev. E. S. Hilliard proposed, with much pleasure, the following resolution : " That the best thanks of the Council be, and are hereby accorded, to the Committee of Distribution for the care they have bestowed upon the preparation of the awards." The report itself constituted a proof of the great trouble the Distribution Committee had to take before such a rendering could be presented.

The Rev. Hardy Harwood seconded, remarking that during his long connection with the Council he had been very greatly impressed by the impartiality shown; and whenever the Council's decisions and allotments had been challenged the evidence showed that both sides had been fully looked into as a matter of course, and the decision was fully justified.

The Chairman, in putting the motion, reminded the Council that the awards were determined actuarially, so that favouritism was impossible.

The resolution was carried.

Canon Pearce proposed : " That the thanks of the Council be, and are hereby given, to the editors of newspapers who have pleaded the needs of the hospitals and advocated the cause of this Fund." It was scarcely necessary to say what a debt the Fund owed to the press, and that obligation was likely to be enhanced by the insistence of journals on the voluntary principle on which the Fund stood. The nursing conference, to which the Chairman referred, seemed to turn largely on the two principles whether hospital and nursing work was in future to be done at the expense of the taxes of the State or whether the voluntary principle was to be encouraged, at all events in partnership with the State. This Fund stood for the continuance of the voluntary principle, and by insisting upon that principle the press were rendering the best possible service.

The resolution was carried unanimously.

Lord Meath proposed : " That the cordial thanks of the Council be, and are hereby given, to the Right Hon. Sir Thomas Vansittart Bowater, Lord Mayor, who, as President and Treasurer, has devoted his valuable time and influence to the well-being of the Fund." The Lord Mayor and his various predecessors had done an immense amount for the Fund, otherwise he did not think it could have been so successful.

Prebendary Ingram seconded, and it was carried.

Lord Meath proposed a vote of thanks to Sir William Church for having presided.

Dr. E. Parker Young, in seconding, paid a tribute to Sir William Church's work on the Distribution Committee and on the Council generally.

The resolution was passed without dissent, and was duly acknowledged.

